# Reconstruction and Analysis of Human Kidney-Specific Metabolic Network Based on Omics Data

**DOI:** 10.1155/2013/187509

**Published:** 2013-10-05

**Authors:** Ai-Di Zhang, Shao-Xing Dai, Jing-Fei Huang

**Affiliations:** ^1^State Key Laboratory of Genetic Resources and Evolution, Kunming Institute of Zoology, Chinese Academy of Sciences, Kunming 650223, China; ^2^Graduate School of the Chinese Academy of Sciences, Kunming 650223, China; ^3^Kunming Institute of Zoology, Chinese University of Hongkong Joint Research Center for Bio-resources and Human Disease Mechanisms, Kunming 650223, China

## Abstract

With the advent of the high-throughput data production, recent studies of tissue-specific metabolic networks have largely advanced our understanding of the metabolic basis of various physiological and pathological processes. However, for kidney, which plays an essential role in the body, the available kidney-specific model remains incomplete. This paper reports the reconstruction and characterization of the human kidney metabolic network based on transcriptome and proteome data. In silico simulations revealed that house-keeping genes were more essential than kidney-specific genes in maintaining kidney metabolism. Importantly, a total of 267 potential metabolic biomarkers for kidney-related diseases were successfully explored using this model. Furthermore, we found that the discrepancies in metabolic processes of different tissues are directly corresponding to tissue's functions. Finally, the phenotypes of the differentially expressed genes in diabetic kidney disease were characterized, suggesting that these genes may affect disease development through altering kidney metabolism. Thus, the human kidney-specific model constructed in this study may provide valuable information for the metabolism of kidney and offer excellent insights into complex kidney diseases.

## 1. Introduction

Metabolic syndrome (MetS) is a complex disorder characterized by extensive metabolic changes in the patients such as the levels of glucose, cholesterol, and uric acid, [1]. People with MetS are at increased risk of various diseases. Observational studies revealed that MetS has a 55 percent increased risk of kidney problems, especially significant alterations to the structure and functions of kidney [2, 3]. Thus, metabolism has been a field of study in modern medicine. With the advent of the high-throughput data production, reconstruction and analysis of metabolic network could complement experimental investigations into various aspects of human disease and provide insight into pathophysiology.

The global human metabolic network, termed Recon 1 [5], has been constructed allowing the comprehensive analysis of human metabolism and disease. However, this generic network only provides a global genome-scale description of human metabolic capabilities without consideration of tissue-specific information. Unlike Escherichia coli and Saccharomyces cerevisiae, human is a multicellular, multiorgans organism, and different tissues have different metabolic objectives and functions. Particular tissue's cells in the human body do not utilize all the metabolic components encoded by the whole genome. In order to mimic in vivo environment, tissue-specific or cell-specific metabolic network will be essential. Several preliminary tissue-specific or cell-specific metabolic networks have been reconstructed and proved to facilitate better understanding of human metabolism in detail [6–9]. Kidney plays a profound role in regulating many important body functions, and it is also an important source of several important hormones. Nowadays, chronic kidney disease (CKD) is becoming a worldwide public health problem and proved to be a risk factor for cardiovascular disease [10]. These issues highlight the importance of constructing a kidney-specific metabolic network, which will provide insight into physiological and pathological processes in the kidney.

To elucidate and understand metabolic genotype-phenotype relationship in human kidney, here a comprehensive human kidney-specific metabolic network was reconstructed by integrating gene expression data from the Gene Expression Omnibus (GEO) [11] and proteome data contained in the Human Protein Atlas (HPA) [12]. We applied model-building algorithm (MBA) [6, 13] by using Recon 1 as a template, the algorithm MBA can automatically select only those genes that are relevant to the target tissue out of the generic model based on the literature and multiple omics data. After reconstruction of the human kidney-specific metabolic network, a series of subsequent analyses were performed to validate and explore the utility of this model. Firstly, we analyzed the gene essentiality by classifying all genes of this model into kidney-specific (KS) and house-keeping (HK) types. Secondly, we detected new metabolite biomarkers for various subtypes of kidney disease. Then, a comparative analysis among the metabolic networks of kidney and other tissues was performed, which allowed identification of tissue-specific metabolic features and may be helpful in understanding the discrepancies of tissue-specific functions. Finally, we used human diabetic kidney disease (DKD) as a case to demonstrate the utility of the kidney model by detecting the influence of differentially expressed genes (DEG) on kidney metabolism. In summary, this model is a comprehensive description of the metabolism of human kidney and will allow for tissue-level simulations to achieve a better understanding of kidney-related disorders.

## 2. Materials and Methods

### 2.1. Data Preparation and Filtering

Tissue specificity information was primarily based on protein abundance from the online database. Firstly, we retrieved kidney specific proteome from HPA [12], which gave an in-depth detailed quantitative proteome data in the form of cell types in each tissue. We adopted two types of cell (including glomeruli and tubules cell) to represent kidney tissue. Here the genes/proteins with positive immunohistochemical signals (weak, moderate, or strong) were considered active in the corresponding cell type. Furthermore, for each gene, if its protein evidence summary score was high, medium, or low, we still regarded this protein as active in this cell type. The protein evidence summary score was calculated based on three parameters: UniProt protein existence (UniProt evidence), transcript profiling categories (RNA evidence), and a Protein Atlas antibody based score (HPA evidence). Finally, a total of 12,344 genes were identified in the above two types of cell. These data were used as the main evidence for assessing the presence or absence of metabolic genes in the kidney. However, current proteomic analysis is somewhat limited due to biochemical and technological challenges, such as protein degradation, low-abundance, and lack of advanced analytical methods. Thus, a relatively large number of genes expressed in tissues cannot be detected in proteome study [14].

Transcriptome data of kidney was used as an extra evidence to provide complementary insights into expression patterns at the RNA level. We obtained human microarray expression data GSE1133 [15] from the GEO of NCBI. This microarray dataset was a comprehensive transcriptome description of human and mouse on a genome-scale and was also used as the expression resource for BioGPS [16], which is a free extensible and customizable gene annotation portal. We retrieved human kidney tissue expression samples (GSM18955, GSM18956) and applied gcrma normalization method for expression level process. On a chip, each gene is represented by one to several probes. The expression level detected by each probe set was obtained as the signal intensity (*S*). We averaged *S* values among replicates to compile an extensive set of comparable data. And an expression data matrix was produced, where a row represents expression levels of a gene while a column is the expression pool in a sample. Then, we filtered out genes with absolute values less than 10th percentile (default value) using MATLAB function genelowvalfilt.m. We considered the remaining genes above the value as active [17–19]; then 11,518 genes were identified in human kidney.

The detected genes from HPA and GEO were separately mapped to the enzyme-encoding genes in the generic metabolic model (1794 in total). If gene transcripts and proteins were both identified or only proteins were identified, we think they are actually active in human kidney. If only gene transcripts were identified (419 genes), the expression activities of these genes were further validated by using another transcriptome dataset (GSE11560) [20]. The dataset was used to measure gene expression levels in livers, kidneys, and hearts from humans, chimpanzees, and rhesus macaques using a novel multispecies microarray [20]. We treated this dataset following the same criteria and found that all the 419 genes were also detected in this human kidney. Finally, we discarded those genes that were not identified in both datasets (proteome data and transcriptome data).

### 2.2. Model Reconstruction and Simulations

The genome-scale human metabolic network model (Recon 1) [5], which contains 1,794 genes, 1,903 metabolites, and 2,942 reactions, was used as a template for the reconstruction of kidney-specific metabolic model. Heretofore, several algorithms aiming to reconstruct a tissue or condition-specific metabolic network from a generic model have been developed [21]. Here, we apply the algorithm MBA by integration with proteome and transcriptome data presented above. This algorithm is aiming to create context-specific subnetwork in a random order based on heuristically pruning the generic human metabolic model. MBA is executed repeatedly for a number of times (1000) with different, random scanning orders. The resulting model is as consistent as possible with the pertaining tissue-specific molecular data sources [6, 13]. The scripts deleteModelGenes and removeRxns were applied to remove a gene and its related reactions only if the removal did not prevent biomass production. We further analyzed the resulting model by using several methods in the COBRA toolbox [22], including flux balance analysis (FBA), minimization of metabolic adjustment analysis (MOMA), and flux balance analysis (FVA).

### 2.3. Gene Essentiality Analysis for Kidney-Specific and House-Keeping Genes

In order to investigate the genetic functions and associations between genes and kidney-related diseases, we categorized genes in the human kidney model into HK genes and KS genes according to previous work [23] which identified human HK genes and tissue-specific genes by using microarray meta-analysis. We mapped these HK and KS genes to our model. Then single gene knockout simulations were performed by changing their associated reaction bounds to zero in the model. In addition, in silico flux variability analysis (FVA) on nonessential gene deletion strains was also performed to assess the consequence of the model after deleting a nonessential gene. The absolute flux span is a measure of the flux range for each reaction. It is calculated as follows:

(1) fi=abs(vmax⁡,i−vmin⁡,i),ri=fi,kfi,w,

where *v*_max⁡,*i*_ and *v*_min⁡,*i*_ represent the maximal flux and minimal flux as determined by FVA. And *r* describes the ratio of knockout strains flux span (*f*_*i*,*k*_) to one of the wild-type strains (*f*_*i*,*w*_).

### 2.4. Prediction of Metabolic Biomarkers

The identification of new biomarkers has proven to be useful in early diagnosis of inborn errors. The potential clinical utility of the metabolic model was previously demonstrated by its ability to predict metabolic biomarkers, and marked correlation was observed between genomic mutations and the altered concentration of metabolites [24, 25]. Here, we applied constraint-based modeling method, a novel computational approach developed by Shlomi et al. [24], to predict metabolic biomarkers for kidney-related diseases in this kidney stoichiometric metabolic model. Firstly, the genes set associated with renal development and function were downloaded from the public resource database, European Bioinformatics Institute (EBI) (http://www.ebi.ac.uk/GOA/kidney) [26]. Then, we performed gene functional annotation for these genes by using DAVID bioinformatics enrichment tools [27, 28] to identify kidney-related disease genes, following the classification criteria of Online Mendelian Inheritance in Man (OMIM database). Finally, constraint-based modeling method was applied to predict novel potential metabolite biomarkers using the list of kidney-related disease genes identified above. Small widespread metabolites, such as hydrogen, ion, and water, were not applied in the further analysis because they are unsuitable for biomarkers. To further validate our prediction, we compared predicted biomarkers alterations with the biomarker data from Human Metabolome Database (HMDB) [29] in several metabolic disorders.

### 2.5. Comparison of Tissue-Specific Models

To systematically analysis tissue-specific metabolic behavior, we compared kidney metabolic network with several other tissue-specific networks from previous work, including heart-specific network and live-specific network [6, 9]. Then different subnetworks were detected corresponding to different tissues by using NetworkAnalyzer [30]. The GO annotation for the genes of these subnetworks was further performed by using BINGO [31], a biological network gene ontology tool, which is implemented as a plugin for Cytoscape [32]. Only categories with a low *P* value (<0.01) were considered as enriched in the network as determined by Hypergeometric statistical test employing the Benjamini and Hochberg false discovery rate correction.

### 2.6. Simulations on Differentially Expressed Genes of Human Diabetic Kidney Disease

This kidney metabolic model provides a useful resource for studying the metabolic basis and molecular mechanism for a variety of human kidney-related diseases. As a demonstration of the utility of models of kidney disease, we used DKD as a case, which is considered a nonimmune-mediated degenerative disease of the glomerulus and recently becoming the primary end-stage renal disease (ESRD) worldwide. We retrieved the 330 overlapping DEG in both glomeruli and tubuli from a recent study [4] which performed a comprehensive gene-expression analysis between DKD samples and the normal counterpart. Significance of DEG was determined using the Fisher's exact test (*P*-value < 0.05) with the Benjamini-Hochberg multiple testing correction and fold change >1.5. We mapped these genes to our constructed metabolic model and identified the upregulated or downregulated genes. Then, we performed FVA to assess the consequence of the model after perturbation. Subsequently, we analyzed the altered metabolic pathway in detail.

## 3. Results

### 3.1. Generation of Kidney-Specific Metabolic Network

**Figure 1:**
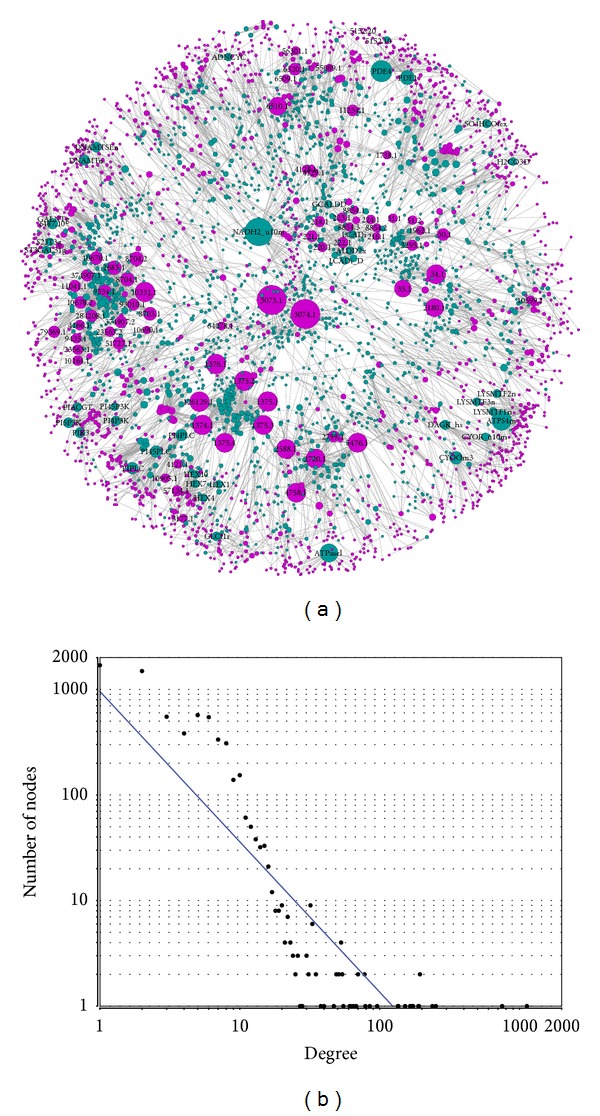
Reconstruction of human kidney-specific metabolic network. (a) illustrates the associations of genes with the linked reactions in the kidney metabolic network. The red circles represent genes (genes with the degree >10 are labeled with Entrez Gene ID), while the blue circles represent the linking reactions, and the lines represent the relationships between genes and reactions. (b) shows the node degrees distribution of the kidney metabolic network.

After applying the MBA method, the resulting kidney model consists of 2904 reactions, 1898 metabolites, and 1776 genes which are mainly enzymes and transporter genes. The kidney model of partially compartmentalization patterns, in SBML format, was generated (Supplementary File: kidney_model_par.xml in Supplementary Material available online at http://dx.doi.org/10.1155/2013/187509). The network visualization can be explored interactively using the freely available Cytoscape software.  Figure 1(a) illustrates gene-reaction associations in the kidney metabolic network; it demonstrates that the genes are close to each other and each metabolic reaction is associated with one or more enzymes. The metabolic processes are largely involved in energy metabolism, extracellular transport, glycerophospholipid metabolism, heme synthesis, and nitrogen and lipid metabolism. The subcellular localization was ignored as the same metabolite could be localized in different cellular compartments being linked by transport reactions. By analyzing the network, we found that the degrees of this network follow the power-law distribution (Figure 1(b)), suggesting that most genes are involved only a few reactions while only a small number of genes participate in the generation of a large number of products.

### 3.2. Functional Characteristics of Kidney-Specific Genes and House-Keeping Genes

**Figure 2:**
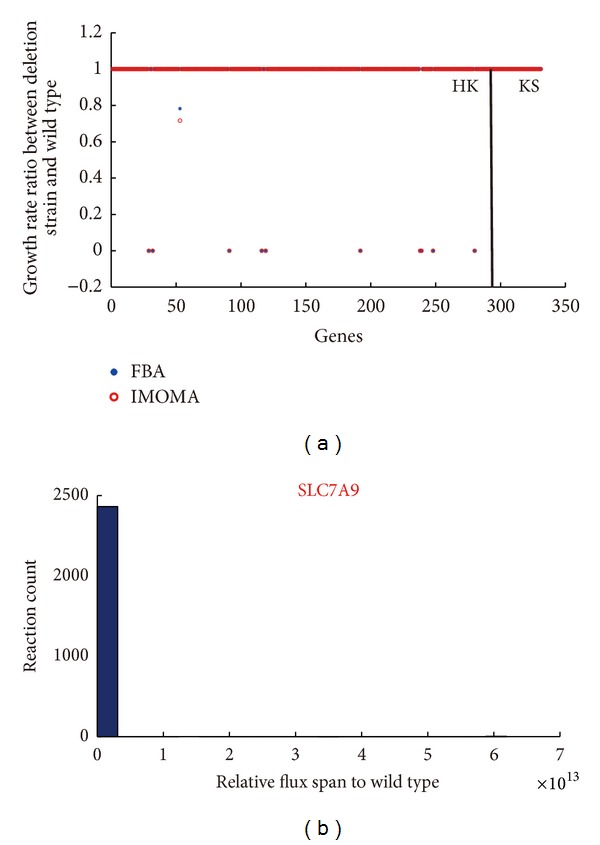
The functional characteristics of KS and HK genes in the kidney-specific metabolic model. (a) shows the distribution of predicted relative growth rates of HK and KS genes using both FBA (blue star) and linear MOMA (red circle) methods. The *y* axis represents growth rate ratio between deletion strain to knockout strain, and the *x* axis represents the 297 HK genes and 34 KS genes. (b) shows a typical example of effects of KS gene mutant strains on the network flexibility. In the mutant strain of Solute carrier family 7 member 9 (SLC7A9), the majority (99.51%) of the metabolic reactions do not change flux span compared to wild-type strain while 12 reactions have much higher (*r* > 2) flux span.

We performed gene essentiality analysis by categorizing genes in the human kidney metabolic model into HK genes and KS genes. We totally retrieved 55 KS genes and 2064 HK genes from previous study [33], then we mapped these genes to the kidney model. Finally, only 24 KS and 233 HK genes are present in our kidney model, the other 31 genes were discarded for they are not the component genes in kidney metabolism. If we take the information of cellular compartments into consideration, then the numbers of above genes turned out to be 34 (KS genes) and (297 HK genes). Single gene deletion experiments were performed with these genes by using both FBA and linear MOMA methods to characterize the gene deletion phenotypes. The distribution of predicted relative growth rates of knockout strains to wild-type strains for all the mapped gene deletions in the kidney model was shown in Figure 2(a). All the 34 KS genes are nonlethal, while out of 297 HK genes, 10 were considered lethal and one resulted in reduced maximal growth rate. It suggests that HK genes are mainly involved in fundamental cellular functions and knockingout these genes may cause metabolism alterations, thus result in lower growth rate or lethality. In contrast, the functions of KS genes can be complemented by other members in the same gene family, such as ATPase alpha/beta chains family and solute carrier family. The latter is a large family and transport succinate and other Krebs cycle intermediates and play an important role in the handling of citrate in kidney. Consequently, their single gene mutations do not lead to lethality in human. Furthermore, these KS genes may play a role in kidney's particular function (such as sodium ion and inorganic anion transport) other than the fundamental cell activity (Figure  S1). Through the analysis of metabolic networks, we found that these KS genes mainly belong to transport subsystem or amino acid metabolism subsystem. We listed the detailed information about these genes and the involved reactions in Table  S2.

Additionally, we further detected the alteration of the metabolic network flexibility after knockingout of the nonessential KS genes. The 24 KS genes were included in this analysis. FVA was performed on these KS genes knockout strains. Figure  S2 demonstrates that flux spans of the metabolic reactions less likely to fluctuate for all the 24 KS genes. Flux spans of metabolic reactions were detected to fluctuate only for eight KS genes, just take solute carrier family 7 member 9 (SLC7A9) mutant strains as an example (Figure 2(b)). The result shows that the majority (99.51%) of the metabolic reactions flux span do not change compared to wild-type strains, while 12 reactions have much higher (*r* > 2) flux span. These reactions are relevant to various catalytic activities, like dehydrodolichol phosphate phosphatase, dimethylallyl transtransferase, diphosphomevalonate decarboxylase and so on. The other 16 KS gene mutant strains have no effect on the network flexibility.

### 3.3. Prediction of Specific Metabolic Biomarkers for Kidney-Related Diseases

Biomarkers have a significant impact on the care of patients and are urgently needed for advancing diagnostics, prognostics, and treatment of disease. Recently, the generic model was successfully used to predict changes in metabolite concentrations caused by genomic mutations [24]. Advances in omic profiling technologies, especially biofluid metabolomics, offer the possibility of detecting early diagnostic biomarkers and pathways activated in disease or associated with disease conditions [33]. Previous study has performed a series of comparative statistical analysis between liver-specific metabolic model and generic model in the ability of prediction performance [6]; it suggests that tissue-specific model serves as a much better basis for predicting tissue alterations and can better predict biomarker changes in genetic metabolic disorders than the generic human model accurately. To provide the fundament for the kidney-related diseases prevention and diagnosis, we performed the analysis here focusing on metabolic disorders that arise from mutations in renal metabolic genes. According to procedure of material (see Section 2), a total of 552 kidney-related disease genes were retrieved, and 122 kidney genes were mapped onto the kidney model. These genes have been experimentally or clinically proved to be disease genes. After applying the constraint-based modeling method to our model, we detected a set of 267 metabolites whose concentrations are predicted to be either elevated or reduced due to 50 possible dysfunctional genes (see Table  S1) [34]. Small and widespread metabolites (such as H_2_O, O_2_) were excluded for these molecules which are not suitable for biomarkers.

**Figure 3:**
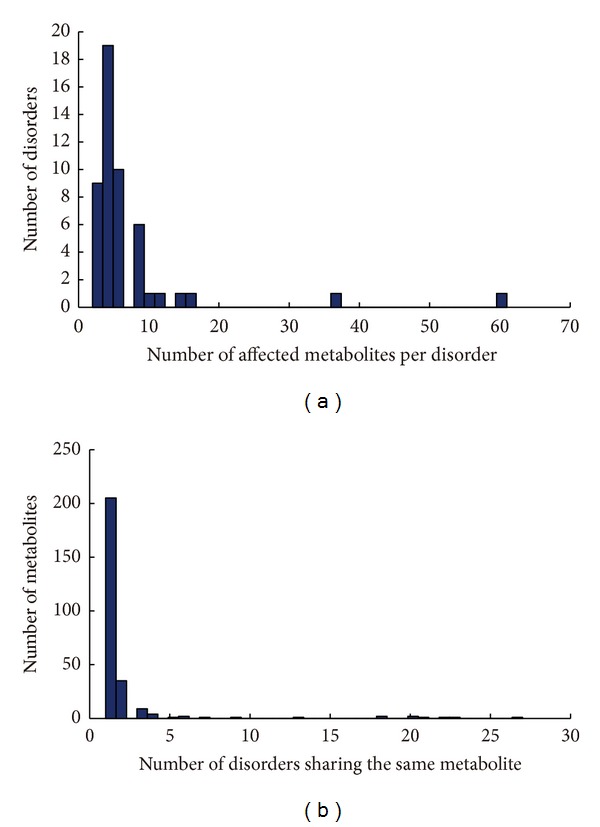
The statistical relationships between the predicted biomarkers and disorders. (a) shows the distribution of predicted metabolic biomarker alteration patterns that are jointly shared by a number of disorders. The majority of disorders (78%) are predicted to have very few biomarkers (≤6). (b) shows the distribution of the number of the predicted alterations among the disorders recorded in OMIM database.

In order to prove the specificity of our biomarker prediction, the relationships between disease types and the predicted biomarkers were further studied.  Figure 3(a) shows that the majority of disorders (78%) are predicted to have very few biomarkers (≤6), whereas up to 90% of the disorders have ≤10 biomarkers. Meanwhile, the various disorders tend to have different sets of biomarkers, for example, only in a few cases, the same set of biomarkers correspond to more than five diseases (Figure 3(b)). The results suggest that these biomarkers can be effectively used for the early diagnosis of kidney metabolic disorders. Furthermore, to consolidate our prediction, we compared predicted biomarkers alterations with biomarker data from Human Metabolome Database (HMDB) [29] for several metabolic disorders. Beyond all doubt, our predictions are shown to significantly correlate with known disease biomarkers from HMDB. Moreover, we discovered many novel potential biomarkers. Here we take one disorder Hawkinsinuria as an example to support our prediction. Hawkinsinuria is also called 4-Alpha-hydroxyphenylpyruvate hydroxylase deficiency, which is an autosomal dominant metabolic disorder affecting the metabolism of the sulfur amino acidhawkinsin [35]. One small molecule metabolites phpyr [c] (4-Hydroxyphenylpyruvic acid) identified in our study has been deposited in the HMDB database for applications in clinical metabolomics (HMDB00707). However, several other novel potential metabolite biomarkers we predicted are not present in HMDB for this disease type. These biomarkers can be used as complement for the HMDB database for this disease. Overall, our predictions are credible and reliable. These biomarkers would be useful to augment the information obtained from traditional indicators and illuminate disease mechanisms. Notably, before these biomarkers are incorporated into clinical practice, rigorous experimental validation and clinical evaluation will be needed.

### 3.4. Kidney Metabolic Genes Are Largely Involved in Amine Metabolic Process

**Figure 4:**
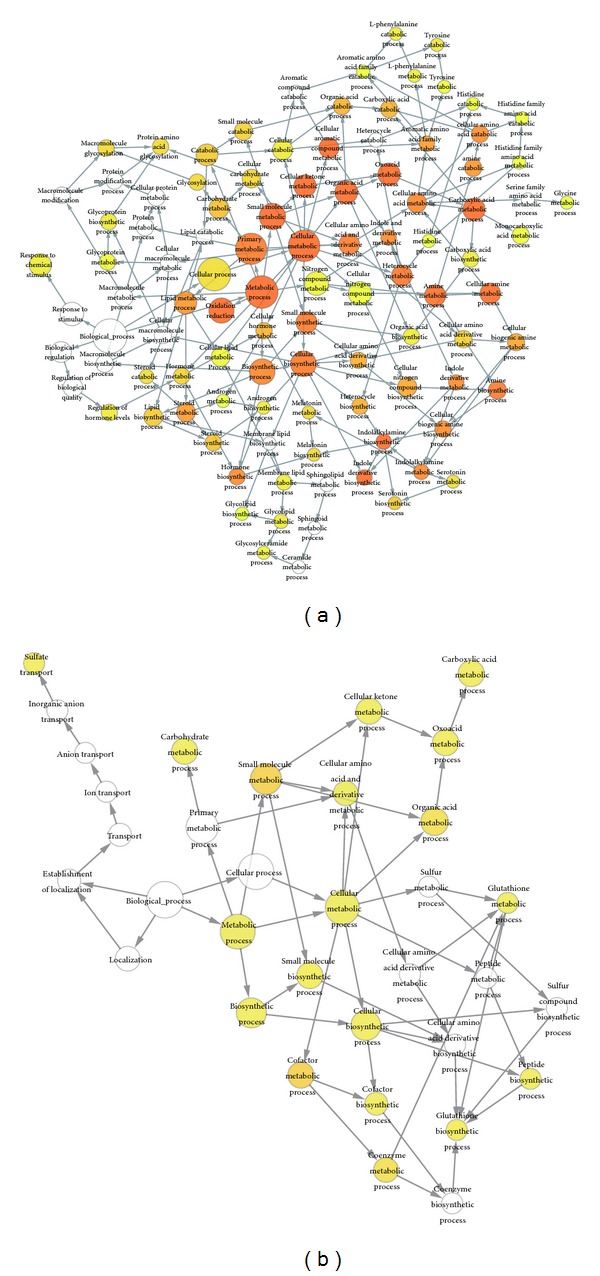
Biological processes enrichment analysis for PDG in comparison of kidney-specific metabolic network to heart-specific metabolic network. (a) shows the cellular metabolic processes overrepresented by kidney-PDG compared to the model of heart; it indicates that the kidney metabolic genes are largely involved in various processes, like amine metabolic process, indolalkylamine biosynthetic process, and hormone biosynthetic process. (b) shows the cellular metabolic processes overrepresented by heart-PDG compared to the model of kidney, it indicates that the heart metabolic genes are largely involved in other cellular process, such as cofactor metabolic process, coenzyme metabolic process, and glutathione biosynthetic process. GO annotation was performed by using BINGO. The yellow to orange color of the circles represents enriched GO categories, and the darkness of color is proportional to the significance level; the size of circle is proportional to the number of gene cluster annotated to that node. Only categories with a low *P* value (<0.01) were considered as enriched in the network. *P* value is determined by Hypergeometric statistical test employing the Benjamini and Hochberg false discovery rate correction.

Tissues execute their functions via different gene sets and metabolic pathways, so tissues may show characteristic metabolic features that make them different from other tissues. Thus, comparing the components of their metabolic models is essential for understanding the tissue-specific metabolic behavior. We compared our constructed kidney metabolic model with two other tissue-specific models obtained from previous work [6, 9], including heart specific model and liver specific model. Then four different small subnetworks, consisting of genes, reactions and metabolites, were generated corresponding to different tissues. We extracted the above genes (named Pair-Different-Genes, PDG) and performed GO enrichment analysis, respectively. We found that different metabolic processes were detected corresponding to different tissues. Here we only elaborated the comparison of kidney and heart in detail. The cellular processes overrepresented by PDG of kidney comparing with those of heart are shown in Figure 4(a). The kidney-PDG were largely involved in several processes, like, indolalkylamine biosynthetic process, hormone biosynthetic process and amine metabolic process, which is important for kidney to filter and eliminate the byproducts of metabolism and regulate many important body functions, especially the urea excretion functions during nitrogen metabolism process. Beyond these processes, other metabolic processes were also found to be significantly enriched in kidney, including oxidation reduction, cellular aromatic compound metabolic processes, cellular ketone metabolic process and the generation of precursor metabolites and energy. The top fifteen overrepresented metabolic processes in kidney with *P* value < 0.01 are listed in Table 1. Consistent with the above finding, for heart metabolic model, its heart-PDG were involved in other cellular processes (Figure 4(b)), including cofactor metabolic process, coenzyme metabolic process, and glutathione biosynthetic process. These processes were corresponding to heart's function of working like a pumping machine to provide the power needed for life. Similar results for comparison between metabolic modes of kidney and liver are also shown in Figure  S3. The detailed information about gene clusters and the corresponding significantly enriched GO categories can be found in Table  S3.

### 3.5. Flux Variability Analysis of Differentially Expressed Genes for Human Diabetic Kidney Disease

**Figure 5:**
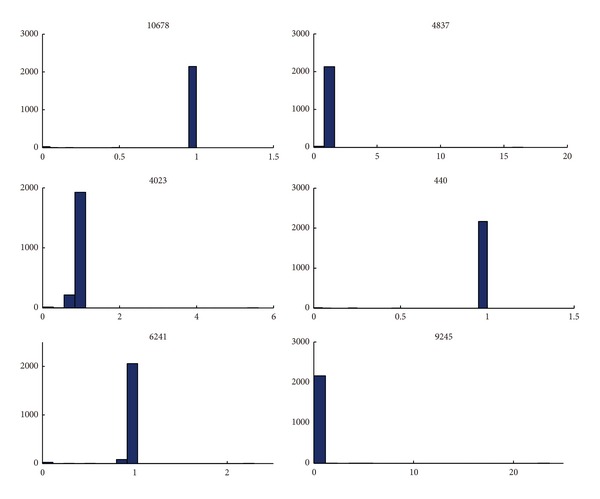
The flux variability analysis of DEG in DKD. The mutation effects of six DEG on the metabolic network flexibility are shown. It indicates that the flux spans of the metabolic reactions evidently fluctuate for the six genes, especially LPL (Entrez gene ID: 4023). The *y* axis represents reaction count, and the *x* axis represents the flux span ratio of knockout strains to wild-type strains. The genes are represented by Entrez gene ID as shown in Table 2.

To further investigate how metabolic genes affect the disease process in human, we illustrated DKD as one case to illuminate this question. We obtained the DEG from a recent DKD study between the disease samples and the normal counterpart. The expression levels of these genes demonstrate remarkable change, revealing that they play a key role in DKD by affecting the involved pathways [4]. Totally 24 of 330 DEG genes mapped to our constructed kidney model. FVA were performed to characterize the gene deletion phenotypes. For most of the genes, the flux spans of their reactions were demonstrated fluctuated distinctly. We listed several genes and their related information in Table 2, and the corresponding FVA analysis results were demonstrated in Figure 5.

In the case of one gene, LPL (lipoprotein lipase; Entrez ID: 4023), which functions as a homodimer, has the dual functions of triglyceride hydrolase and bridging factor for receptor-mediated lipoprotein uptake. The LPL deficiency often leads to in type I hyperlipoproteinemia [36]. In LPL gene deletion strain, we found that dozens of reactions change their flux span compared to wild-type strain, these reactions can be classified into three groups. Type I reactions have much higher flux span, such as EX_crn (*r* > 5), which is involved in Carnitine shuttle subsystem. In contrast, type II reactions have lower flux span, such as GLYCt, PEROXx and FAOXC11 (0 < *r* < 0.5). The flux span of type III reactions reach to zero, including ARTCOAL3 and DEDOLR_U. These reactions are known to be associated with glycerol transport, Fatty acid oxidation, peroxisome, N-Glycan Biosynthesis, R Group Synthesis and Triacylglycerol Synthesis. Consistently, we found that the expression of LPL gene showed severed decreased sharply (9-fold) between healthy and DKD glomeruli. Our results suggest that LPL may play an important role in the DKD development process owing to metabolic deficiencies. These metabolic DEG genes are involved various catalytic processes, and mutations of them will result in serious diseases or lethality. The results presented here coupled with previous gene-expression studies further prove the importance of these genes and provide important insight into the underlying DKD mechanism. 

As we know, many diseases are characterized by extensive metabolic changes in the body. In the case of DKD, specific metabolically driven and glucose dependent pathways were reported activated in diabetic renal tissues [37]. Recently, metabolic analysis technologies, such as FVA, could offer excellent insights into the development and progression mechanism of complex diseases.

## 4. Discussion

Among the different types of biological networks, the metabolic network is of special interest. First of all, it is the most complete and reliable network due to decades of biochemical research so far. Secondly, metabolic network can integrate the different types of experimental omics data, such as transcriptomics for genes, proteomics for enzymes, and metabolomics for metabolites. Furthermore, the health and disease states of the human can be described more meaningfully by the metabolic state of human specific type of cells, tissues or organs. 

As one of the most important tissues, kidney plays an essential part in regulating many important body functions. Therefore, it would be valuable to construct a complete and reliable human kidney-specific metabolic network to obtain a better understanding of the relationship between human kidney metabolism and diseases. In this study, we reconstructed a comprehensive kidney-specific metabolic network by integrating transcriptome and proteome data using an algorithm (MBA) with Recon 1 as a template. Then we performed a series of analysis to elucidate the metabolic genotype-phenotype relationship in human kidney. Firstly, we performed gene essentiality analysis by categorizing genes into HK genes and KS genes. The results indicate that HK genes are always involved in fundamental cellular functions, knockingout of these genes may cause metabolism alterations resulting in lower growth rate or lethality. In contrast, the KS genes are mainly relevant to tissue-specific functions and diseases. And the functions of KS genes can be complemented by other members in the same gene family. Consequently, their mutations do not lead to lethality in human. In summary, our study provided important insight into studies of genetic functions of different types of genes.

As stated in the introduction, the human metabolism is more closely related to human diseases compared with genes and proteins. Many human diseases are characterized by an abnormal metabolic state, such as a high glucose concentration in the blood of diabetes patients and high urine amino acid level resulting from liver or renal disorders. For a long time, the utility of metabolites from blood or urine samples as biomarkers has been proved effective for diagnosing diseases. Several hundred diseases are characterized by metabolic syndrome, which is mainly caused by a deficiency of enzymes and the subsequent altered concentration of essential metabolites. In order to give an in-depth insight into disease focusing on the relationship between diseases and kidney metabolism, we obtained the disorder-gene association information from the OMIM and detected new metabolite biomarkers for kidney-related diseases. And the subsequent analysis suggests that our predicted biomarkers are suitable for early diagnosing of different kidney disorders, because that various disorders tend to have different sets of biomarkers. Additionally, the results are consistent with the existing experimental and clinical data. On the basis of our results, it would be worthwhile to further examine the predicted biomarkers for disease diagnosis. It should also be noted that not all kidney disease genes were included in our study, and here we only analyzed the kidney disease related enzymes. Our finding provides the fundament for a subsequent disease prevention and diagnosis.

Furthermore, we compared our constructed kidney-specific metabolic model with other two tissue-specific models aiming to find some tissue-specific metabolic behaviors in which the involved genes have strong evidence supporting their function in the respective tissue. The results show that the kidney metabolic genes were largely involved in amine metabolic process, indolalkylamine biosynthetic process, hormone biosynthetic process, and so on. It could provide an explanation of how kidney executing its functions, such as filtering the byproducts to form urine and regulating important body functions. In brief, tissues execute their functions via different gene sets and metabolic pathways. Advances in microarray and RNA-seq technologies allow the systemic analysis and characterization of expression level alterations of genes during the disease process. As a demonstration of the utility of our model in kidney disease research, we took DKD as an example and performed gene essentiality and flux variability analysis for DEG to characterize the gene deletion phenotypes. We found that dozens of reactions changed their flux spans compared to wild-type strains, especially in the LPL gene deletion strain. It suggests that these DEG genes may be possible to affect disease development via altering metabolic flux distributions. These findings could offer excellent insights into metabolism research and illuminate DKD disease mechanisms.

Metabolic network has been proved to be an effective way to study the molecular mechanism of disease occurrence and development. We measured the properties of the drug targets and explored the kidney model to detect potential targets for kidney-related diseases. The GO enrichment analysis shows that these selective drug targets tend to be distributed in the various pathways, including G-protein coupled peptide receptor activity, protein phosphatase binding, growth factor binding, and hormone receptor binding (see Figure  S4). The high quality human metabolic network will help to identify more enzyme targets through systematic analysis of the metabolic network. In a recent paper, several examples have shown that enzyme drug targets were found by using metabolic control analysis [38]. Theoretically, potential drug targets and known drug targets should respond similarly to the exogenous drugs to reach the expected therapeutic effects. Then, based on the known enzyme targets in one metabolic pathway, we can approach some better results by targeting other enzymes to get the expected outcome. Applying FVA on all genes in the kidney model, in the case of HMG-CoA reductase (EC 1.1.1.34), we found that several other enzymes have the same flux alterations (Table  S4); these enzymes may be the potential drug targets similar to HMG-CoA reductase.

In the case of the species evolution, it is necessary to compare the kidney-specific metabolic networks between human beings and common animal models of human diseases, and the information about functionally divergent and conserved metabolic pathways will provide excellent insights into the optimal modeling of human kidney disease.

## 5. Conclusion

In this paper, we constructed a human kidney-specific metabolic model by integrating transcriptome and proteome data. Based on the resulting model, a series of metabolic simulation indicate that HK genes are more essential than KS genes in terms of kidney metabolism. Subsequently, a set of 267 potential metabolite biomarkers for different kinds of kidney disease has been successfully predicted, and further statistical analysis validated the feasibility of being early disease diagnosis. Tissue-specific networks comparison imply that discrepancies in metabolic processes are corresponding to their functions, such as kidney metabolic genes, which are largely involved in amine metabolic process. We further analyzed DEG detected in DKD, and the metabolism alterations were detected; it suggests that these genes may affect disease development through altering metabolic flux distributions. The human kidney-specific model provides valuable information for the studies of urinary system activity and development of kidney disease.

## Supplementary Material

Supplementary Figure S1: Is about GO enrichment analysis of the 24 nonessential KS genes.
Supplementary Figure S2: Is about the flux variability analysis result of metabolic network for the 24 nonessential KS genes.
Supplementary Figure S3: Is about biological processes enrichment analysis for PDG in comparison of kidney-specific metabolic network with liver-specific metabolic network.
Supplementary Figure S4: Is about GO enrichment analysis of drug target genes of kidney-related diseases.
Supplementary Table S1: Is about predicted metabolic biomarkers for kidney-related diseases using kidney metabolic model.
Supplementary Table S2: Is about the involved reactions' information about kidney-specific genes in the kidney-specific metabolic network.
Supplementary Table S3: Is about gene clusters and the corresponding significantly enriched GO categories obtained from BINGO.
Supplementary Table S4: Is about predicted potential drug targets similar to HMGCR.

## Figures and Tables

**Table 1:** Top fifteen overrepresented metabolic processes of kidney-PDG compared with heart.

GO-ID	*P* value	Gene-num	Description
55114	1.78*E* − 15	20	Oxidation reduction
44281	2.92*E* − 12	23	Small molecule metabolic process
6725	2.33*E* − 11	10	Cellular aromatic compound metabolic process
6082	1.37*E* − 10	15	Organic acid metabolic process
8152	2.37*E* − 10	40	Metabolic process
44237	2.95*E* − 10	37	Cellular metabolic process
46219	4.67*E* − 10	4	Indolalkylamine biosynthetic process
42435	4.67*E* − 10	4	Indole derivative biosynthetic process
19752	1.37*E* − 09	14	Carboxylic acid metabolic process
43436	1.37*E* − 09	14	Oxoacid metabolic process
42180	1.88*E* − 09	14	Cellular ketone metabolic process
44106	2.72*E* − 09	11	Cellular amine metabolic process
9308	4.10*E* − 09	12	Amine metabolic process
46483	6.73*E* − 09	11	Heterocycle metabolic process
9309	9.80*E* − 09	7	Amine biosynthetic process

Note. This table lists the top fifteen overrepresented metabolic processes (*P* value < 0.01) in the kidney when compared with heart. This analysis was obtained from BINGO. GO categories were considered as enriched in the network as determined using Hypergeometric statistical test employing the Benjamini and Hochberg false discovery rate correction. The first column is the GO-ID. The second column is the *P* value. The third column gives the number of the gene cluster, and the fourth column shows the description of the corresponding GO-ID.

**Table 2:** Selected DEG in DKD for FVA analysis.

Gene symbol	Entrez gene ID	FC (glomeruli)	FC (tubuli)	Reaction	Gene name
LPL	4023	−9.18501	−4.38411	LPS; LPS2	Lipoprotein lipase

GCNT3	9245	2.12739	1.847147	CORE4GTg	Glucosaminyl (N-acetyl) transferase 3

NNMT	4837	4.353618	7.890573	NNMT	Nicotinamide N-methyltransferase

RRM2	6241	2.526649	3.055954	RNDR1; RNDR2; RNDR3; RNDR4	Ribonucleotidereductase M2 polypeptide

B3GNT1	10678	−2.28274	−1.53357	B3GNT11g	UDP-GlcNAc:betaGal beta-1,3-N-acetylglucosaminyltransferase 1

ASNS	440	−1.53775	2.724576	DEDOLP1_U; EX_asn_L(e); FT	Asparagine synthetase

Note: These DEG were selected from a previous study [4], and their flux spans of metabolic reactions were detected to fluctuate markedly (Figure 5). It suggests the essential role of these genes in DKD process. Knocking out of these genes may cause metabolism alterations and affect the emergence of DKD. The first column is the gene symbol of DEG; the second column is the Entrez gene ID; the third and fourth columns are the fold change value for glomeruli and tubuli cell, respectively; the fifth column shows the representative affected reactions, and the sixth column is gene name.

## Abbreviations

MetS: Metabolic syndrome
MBA: Model-building algorithm
KS: Kidney-specific
HK: House-keeping
DEG: Differentially expressed genes
FBA: Flux balance analysis
FVA: Flux variability analysis
MOMA: Metabolic adjustment analysis
DKD: Diabetic kidney disease
HMDB: Human metabolome database
OMIM: Mendelian inheritance in man
PDG: Pair-different genes.
