# Supplementary material for: Reconstruction and Analysis of Human Kidney-Specific Metabolic Network Based on Omics Data
Source: Biomed Res Int. 2013 Oct 5;2013:187509. doi: 10.1155/2013/187509 (PMC3814056; doi:10.1155/2013/187509)
Supplement: Supplementary file 1 — Supplementary Figure S1: Is about GO enrichment analysis of the 24 nonessential KS genes. Supplementary Figure S2: Is about the flux variability analysis result of metabolic network for the 24 nonessential KS genes. Supplementary Figure S3: Is about biological processes enrichment analysis for PDG in comparison of kidney-specific metabolic network with liver-specific metabolic network. Supplementary Figure S4: Is about GO enrichment analysis of drug target genes of kidney-related diseases. Supplementary Table S1: Is about predicted metabolic biomarkers for kidney-related diseases using kidney metabolic model. Supplementary Table S2: Is about the involved reactions' information about kidney-specific genes in the kidney-specific metabolic network. Supplementary Table S3: Is about gene clusters and the corresponding significantly enriched GO categories obtained from BINGO. Supplementary Table S4: Is about predicted potential drug targets similar to HMGCR. [file 187509.f1.zip › Supplementary.pdf]

Figure S1. GO enrichment analysis of the 24 nonessential KS genes

The figure displays a significant enrichment analysis of GO molecular function of 24 nonessential KS genes. This graph was generated using ClueGO program [1] under the default parameters. Functionally grouped network with terms as nodes linked based on their kappa score level (≥ 0.3), where only the label of the most significant term per function is shown. The node size represents the term enrichment significance, molecular functions are color-coded as indicated in the figure, and edge thickness levels show the relation strength based on the integrated score value between the nodes.

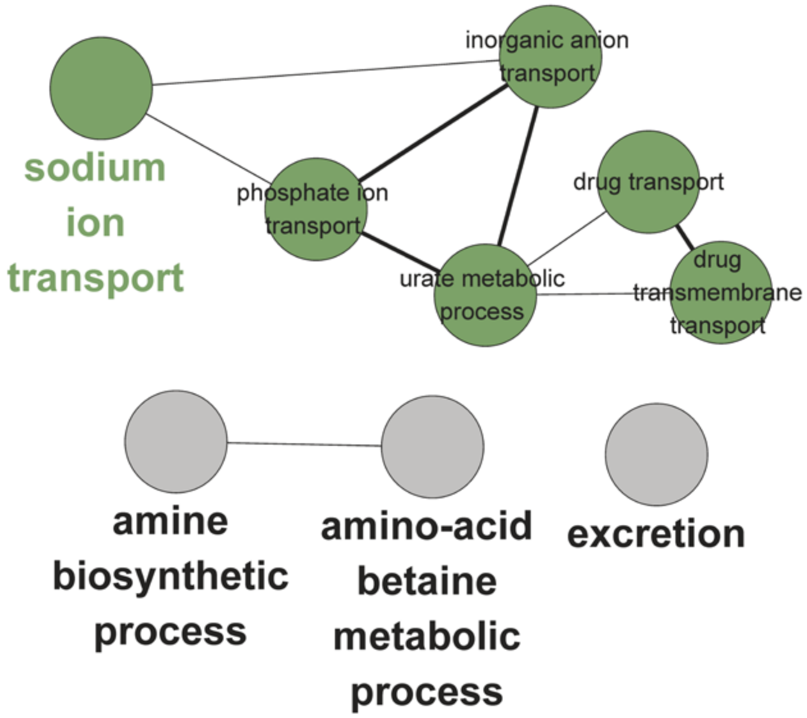

Figure S2. The flux variability analysis result of metabolic network for the 24 nonessential KS genes

The mutation effects of 24 KS gene strains on the metabolic network flexibility are shown in the figure. It demonstrates that flux spans of the metabolic reactions less likely to fluctuate for all the 24 KS genes. For eight KS genes (gene name marked

with a red box), flux spans of their metabolic reactions were detected to fluctuate. The other 16 KS gene mutant strains have no effect on the network flexibility. The y axis represents reaction count, and the x axis represents the flux span ratio of knock-out stains to wild-type strains. The genes are represented by gene symbols.

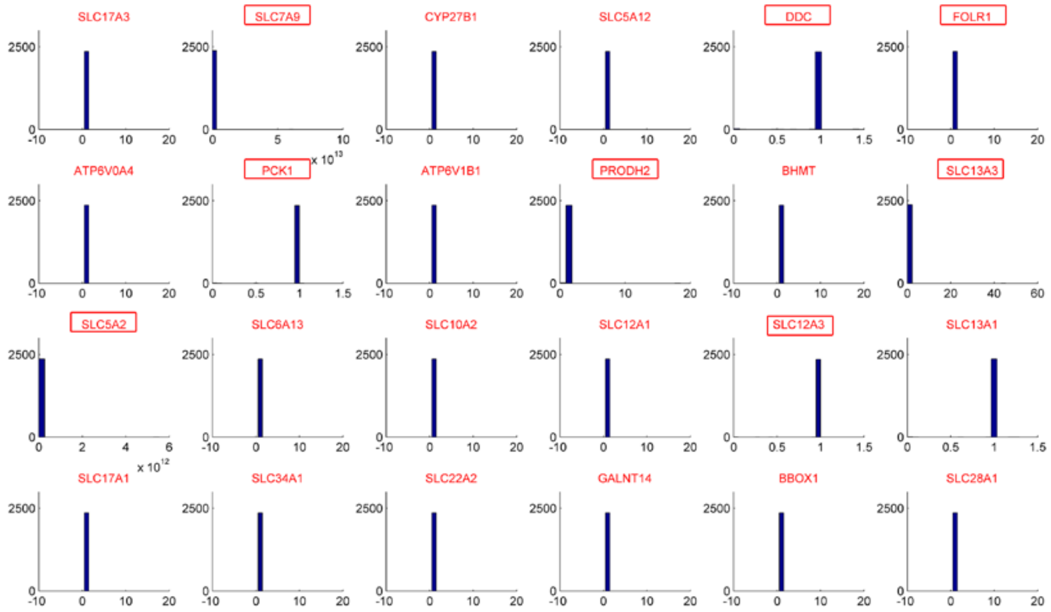

Figure S3. Biological processes enrichment analysis for PDG in comparison of kidney-specific metabolic network with liver-specific metabolic network

Panel (A) shows the cellular metabolic processes overrepresented by kidney-PDG compared with the model of liver, it indicates that the kidney metabolic genes are largely involved in various processes, like amine metabolic process, indolalkylamine biosynthetic process, hormone biosynthetic process and so on. Panel (B) shows the cellular metabolic processes overrepresented by liver-PDG compared with the model of kidney, it indicates that the liver metabolic genes are largely involved in other cellular processes, such as mitochondrial transport, transmembrane transport, small molecule metabolic process and so on. The darkness of color is proportional to the significance level, and the size of the circle is proportional to the number of gene cluster. GO annotation was performed by using BINGO, Only categories with a low p value (<0.01) were considered as enriched in the network as determined by Hypergeometric statistical test employing the Benjamini and Hochberg false

discovery rate correction.

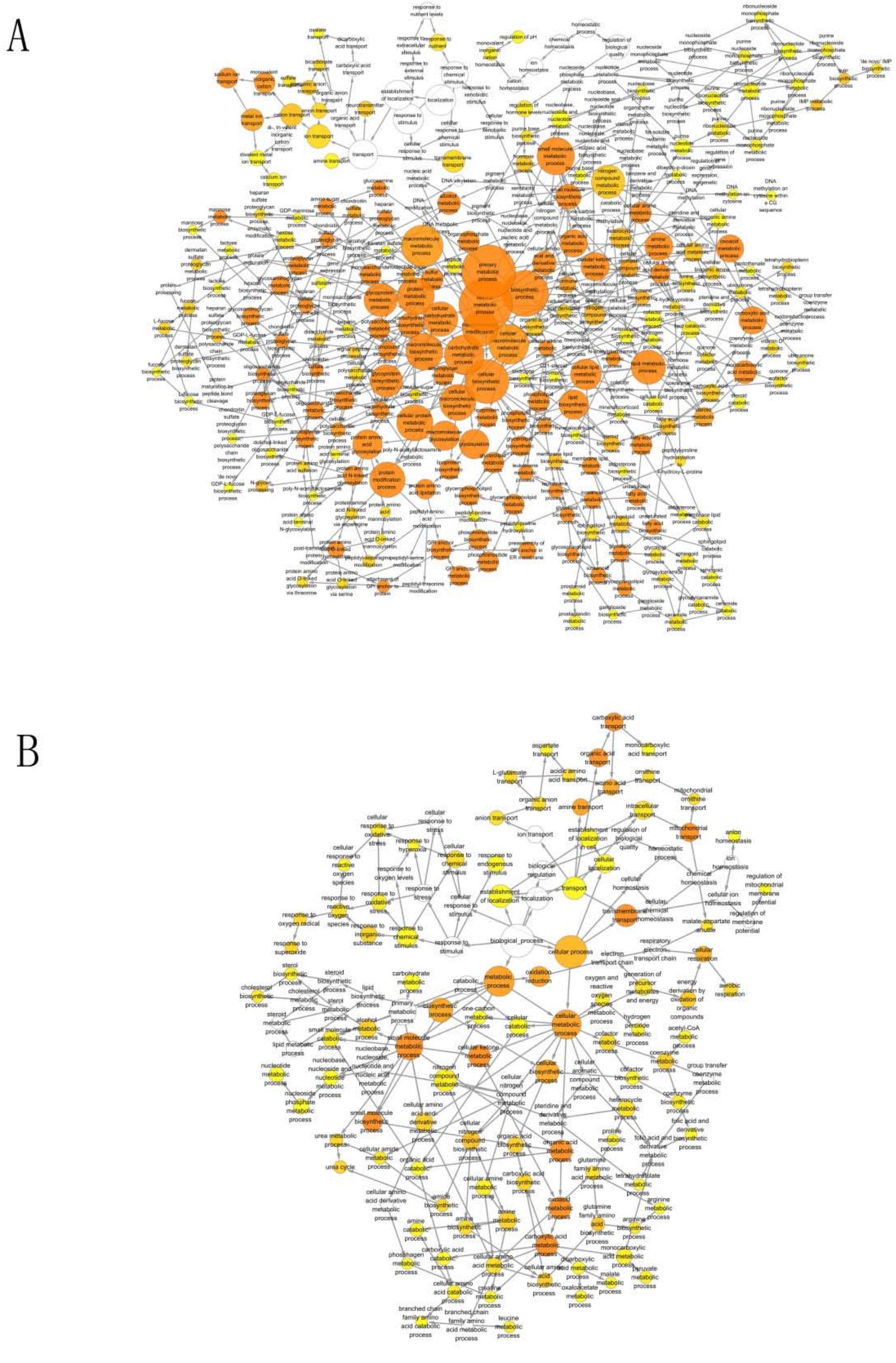

Figure S4 GO enrichment analysis of drug target genes of kidney-related diseases

The chart displays significant enrichment analysis of GO molecular function of 113 drug targets corresponding to 13 types of kidney-related diseases. This information

was retrieved from Thomson Reuters Integrity database. This graph was generated using ClueGO program under the default parameters. Functionally grouped network with terms as nodes linked based on their kappa score level ≥0(3) , where only the label of the most significant term per function is shown. The node size represents the term enrichment significance, molecular functions are color-coded as indicated in the figure, and edge thickness levels show the relation strength based on the integrated score value between the nodes.

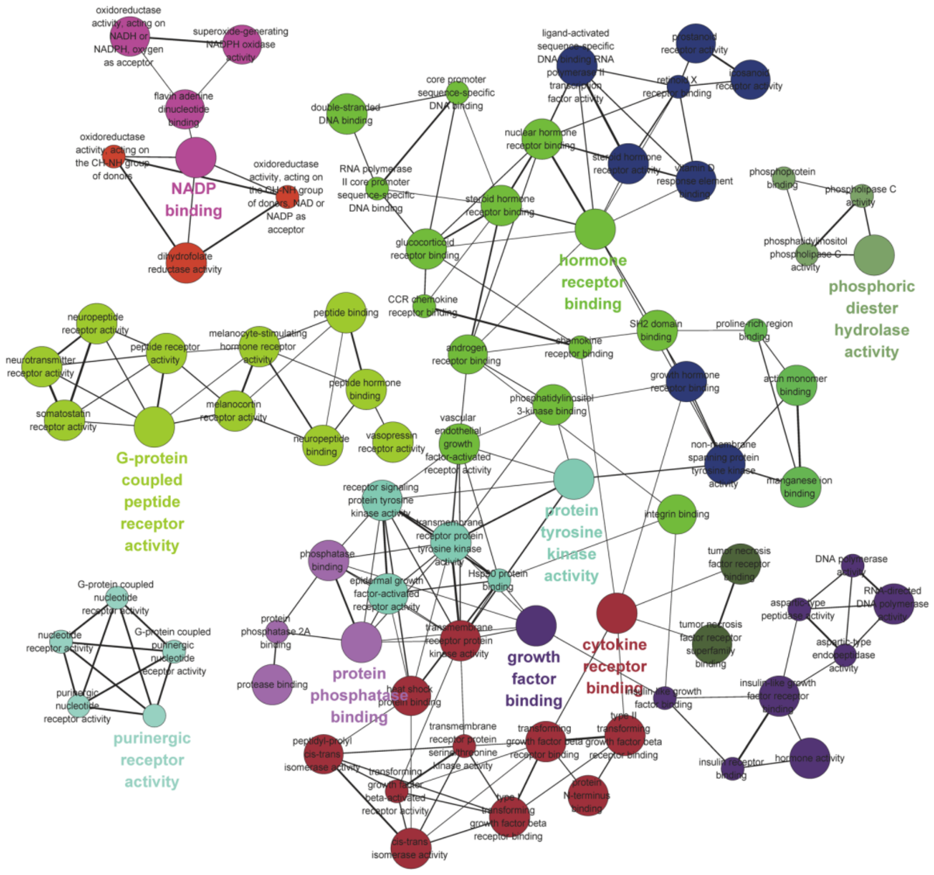

Tables

Table S1.

Predicted metabolic biomarkers for kidney-related diseases using kidney metabolic model are listed. The disease-related genes are according to OMIM disease types, and the predicted biomarkers correspond to Mets records in kidney model file.

Table S2.

The involved reactions' information about kidney-specific genes in the kidney-specific metabolic network is listed.

Table S3.

Gene clusters and the corresponding significantly enriched GO categories obtained from BINGO.

Table S4. Predicted potential drug targets similar to HMGCR (Entrez gene: 3156, 3-hydroxy-3-methylglutaryl-CoA reductase) for treating hyperlipidemia

Entrez GeneID	Gene Symbol	Gene description
10654	PMVK	phosphomevalonate kinase
3157	HMGCS1	3-hydroxy-3-methylglutaryl-CoA synthase 1
4597	MVD	mevalonate (diphospho) decarboxylase
4598	MVK	mevalonate kinase

Reference

1. Bindea, G., et al., *ClueGO: a Cytoscape plug-in to decipher functionally grouped gene ontology and pathway annotation networks.* Bioinformatics, 2009. **25**(8): p. 1091-3.
